# An Orally Administrated Hyaluronan Functionalized Polymeric Hybrid Nanoparticle System for Colon-Specific Drug Delivery

**DOI:** 10.3390/nano9091246

**Published:** 2019-09-02

**Authors:** Niranjan G. Kotla, Orla Burke, Abhay Pandit, Yury Rochev

**Affiliations:** 1 Centre for Research in Medical Devices (CÚRAM), National University of Ireland Galway, Galway H91 W2TY, Ireland (N.G.K.) (O.B.); 2 Sechenov First Moscow State Medical University, Institute for Regenerative Medicine, Moscow 119992, Russia

**Keywords:** colon targeted drug delivery, hyaluronan, polymeric nanoconjugates, curcumin, inflammatory bowel disease, colon cancer

## Abstract

There is a pressing clinical need for advanced colon-specific local drug delivery systems that can provide major advantages in treating diseases associated with the colon, such as inflammatory bowel disease (IBD) and colon cancer. A precise colon targeted drug delivery platform is expected to reduce drug side effects and increase the therapeutic response at the intended disease site locally. In this study, we report the fabrication of hyaluronan (HA) functionalized polymeric hybrid nanoparticulate system (Cur-HA NPs) by using curcumin as a model fluorescent drug. The Cur-HA NPs were about 200–300 nm in size, −51.3 mV overall surface charge after HA functionalization, with 56.0% drug released after 72 h in simulated gastrointestinal fluids. The Cur-HA NPs did not exhibit any cytotoxicity by AlamarBlue, PicoGreen and Live/Dead assays. Following the Cur-HA NPs use on HT-29 monolayer cell cultures demonstrating, the efficacy of HA functionalization increases cellular interaction, uptake when compared to uncoated nanoparticulate system. These findings indicate that HA functionalized nano-hybrid particles are effective in delivering drugs orally to the lower gastrointestinal tract (GIT) in order to treat local colonic diseases.

## 1. Introduction

The site-specific delivery of drugs to the colon provides significant advantages in treating diseases associated with the lower gastrointestinal tract (GIT). An oral delivery system with onsite targetability will enhance therapeutic activity by reducing the associated drug’s systemic side effects, especially with corticosteroids and immunosuppressants. An improved oral, colon-targeted dosage form with local onsite delivery and an appropriate release pattern could be very useful for designing advanced drug delivery technologies for IBD therapy [1,2]. Therefore, advanced drug delivery strategies with site-specificity and local delivery to the inflamed colon or colon cancer tissues for a prolonged period in a sustained manner, and the interaction of the system with epithelial cells is essential for an effective local treatment at the gut wall site [3,4,5].

Current conventional drug delivery capsule/pill strategies (pH, time-dependent, pro-drug, microflora triggered) [6,7,8,9,10,11] are well established in the management and therapy of colonic diseases. However, disadvantages in terms of the inability to target the drugs directly at the diseased tissue, with limited therapeutic efficacy and a high risk of adverse drug reactions make the conventional strategies replaceable [4]. Henceforward, strategies or mechanisms are in the developmental stages during which a payload is delivered by the use of pathophysiological parameters that are directly related to the site of colon inflammation and cancer, which has to be investigated in order to make advanced effective pharmaceutical dosage forms.

There is a large body of literature focused on targeting therapeutics to the lower GIT in order to enhance their therapeutic potential. In recent years, oral pharmaceutical technologies have applied polymer chemistry and nanotechnology strategies to design effective oral dosage forms in an effort to overcome the limitations (such as enhancing the targetability, internalization of drug cargo, local on-site delivery, enhancing the gut epithelial cell interactions) over conventional tablet/pill formulations [4,12]. Polymeric particles are being extensively investigated in this field as an advanced colon-specific drug delivery system that can be specifically modified to target the lower GIT regions with high drug loading, with tunable release rate, and preferential accumulation at the intestinal wall sites are essential to reduce associated drug systemic side effects and minimize the drug excretion from the GI tract [13,14,15,16,17]. A few specific properties of polymeric nanoparticles such as drug encapsulation, drug-polymeric matrix formation, polysaccharide coatings, and ligand functionalization etc., allow them to remain stable in the GIT, protect encapsulated drugs, and modulate drug release properties according to the GIT physiological conditions.

Engineering the nano or microparticles with specific biodegradable, biocompatible polysaccharide coatings (such as guar gum, xanthan gum, chitosan, hyaluronan, pectins, dextrans etc.) can protect the drugs from the stomach acidic environment and improve the targeting ability to the colon with local drug absorption [4,18]. Amongst the synthetic and natural polymers used for nano or microparticle fabrication for oral delivery, poly (lactide-*co*-glycolide) acid (PLGA) is one of the most widely investigated, biocompatible and biodegradable synthetic polymers used for various biomedical applications in humans [19,20]. PLGA nanoparticles appear to be a potential candidate to deliver hydrophobic drug molecules with high entrapment efficiency with controlled release of entrapped medicament [20,21]. However, the PLGA particle system alone is subject to partial degradation in the acidic environment of the stomach and, thus, the coating or functionalization of the drug-PLGA core with polysaccharides to protect the drug degradation in the acidic environment is essential to enhance the therapeutic response at the desired colon site.

Hyaluronan (HA) is a member of glycosaminoglycans (GAGs), which is a major component of the extracellular matrix (ECM) of the skin, gastrointestinal mucosa, joint/disc space, tendons, synovial fluid and vitreous body etc., [22,23,24,25]. HA is one of the ECM components that is located beneath the barrier epithelium, the mucosal layer of the gut wall which facilitates the protection of gut epithelium, acts as a cementing barrier, and prevents gut luminal contents translocation into the systemic circulation [23]. High molecular weight HA has been proven to exhibit anti-inflammatory activity, whereas low molecular weight HA can produce products that can cause inflammation [26]. HA may be administered as a therapeutic agent to help in down-regulation of the intestinal inflammation. Intraperitoneal injection of large molecular weight HA (average ~0.5 × 10^6^ Da) by Zheng and colleagues, protected mice from damage during dextran sodium sulphate (DSS)-induced colitis [27]. The oral delivery of HA has exhibited protective effects on immune-compromised mice from intestinal inflammation through down-regulating pleiotrophin expression via TLR-4 in intestinal epithelial cells [28].

A model fluorescent drug molecule (curcumin) has been used in the current study to prove the drug delivery system’s targetability, release studies in simulated GI fluids, colon epithelial cell interactions, and internalization. Curcumin has also been suggested as a remedy for diseases such as cancer, arthritis, diabetes, inflammatory bowel disease (Crohn’s and colitis) and colon cancer [29,30]. Research investigations on curcumin to bring as a clinical therapeutic drug has been limited due to low potency, poor solubility, poor absorption, low biodistribution, accelerated metabolism and fast elimination [31]. To improve the bioavailability of curcumin synthetic analogues and nanoformulations have been synthesized to increase its therapeutic efficacy.

To our knowledge, little effort has been made in the fabrication of oral nano-drug delivery systems with high molecular weight hyaluronan surface functionalization for an effective cellular internalization following local drug release for colonic specific therapeutic interventions. In this paper, we demonstrate the fabrication, physicochemical characterization of a hyaluronan functionalized polymeric nano drug delivery system which been shown to specifically target any loaded drug cargo to the lower GI regions (in this work we have used curcumin). Polysaccharide coating (chitosan, HA) will protect the payload from acidic stomach environment and deliver the payload in the colonic regions. Furthermore, for the evaluation of nanoparticle cytotoxicity and cell interactions, uptake studies were conducted on colon epithelial-like (HT-29) monolayers.

## 2. Experimental Section

### 2.1. Materials and Reagents

PLGA (Resomer^®^ RG 504H, Poly (d,l-lactide-*co*-glycolide); 50:50, average Mw ~ 38 K–54 K) CAS No: 719900 and Chitosan (average Mw ~ 50,000–190,000 Da), CAS No: 9012-76-4 both purchased from Sigma-Aldrich Pittsburgh, PA, USA. Hyaluronic acid (High Molecular Weight Sodium Hyaluronate 1.2 × 10^6^ Da) purchased from Lifecore Biomedical, Chaska, MN, USA; DMTMM (4-(4.6-Dimethoxy-1,3,5-triazin-2-yl)-4-methylmorpholinium chloride; Mw 276.72), CAS No: 3945-69-5; Pancreatin from porcine pancreas CAS No: 8049-47-6, Pepsin from porcine gastric mucosa, CAS No: 9001-75-6; Polyvinyl alcohol (PVA average 13,000–23,000), CAS No: 9002-89-5 were all purchased from Sigma-Aldrich. Dichloromethane, CAS No: 75-09-02; all other reagents were of analytical grade used in the work.

### 2.2. Fabrication of Curcumin-Loaded Polymeric Nanoparticle Conjugate System

The curcumin loaded HA nanoparticle conjugate system were fabricated using curcumin (4 mg, 8 mg), PLGA (4%, 8% *w*/*v*) dissolved in dichloromethane-DCM (1.5 mL), Ethanol (0.5 mL). There is however no other feasible and rational manner to determine the oral dosage of curcumin at this moment, we have used the aforementioned drug to polymer concentrations to check which batch has attainable surface charge and drug encapsulation, loading efficiencies. This mixture was added dropwise to 1% PVA solution (10 mL) under probe sonication (5 min, pulse on/off: 8 s/2 s, temperature −20 °C and amplitude 30%). The sample was kept under stirring conditions overnight to evaporate DCM, and centrifuged at 13,000 rpm for 5 min. For further coating, 0.2% chitosan and 1% PVA were added to the pellet of the uncoated curcumin loaded PLGA NPs. The mixture was stirred for 4–6 h, and centrifuged at 13,000 rpm for 5 min, and the process was repeated three times. Further, HA (0.2% *w*/*v*), 1.46 mg DMTMM (crosslinking initiator) was added to the chitosan coated NPs (prepared in the previous step). The reaction was stirred overnight as –COOH groups of HA covalently conjugated with chitosan coated NPs (surface amines, –NH_2_). After completion, the reaction mixture was dialysed in distilled water for two days. The nanoparticle solution was then collected from the dialysis bag, lyophilised and stored under vacuum.

### 2.3. Particle Size, Surface Charge Analysis

The particle size and polydispersity index (PdI) of curcumin-loaded nanoparticles were determined by Dynamic Light Scattering (Malvern Zetasizer Nano series Nano-ZS90, Malvern Instruments, Malvern, UK). The sample was diluted and sonicated to ensure a disperse sample. Zeta potential was measured using Malvern Zetasizer Nano series Nano-ZS90, Malvern Instruments, Malvern, UK.

### 2.4. Particle Morphological Analysis

The shape and external morphology of the nanoparticles were analyzed by scanning electron microscopy (SEM) by (S-4700)-Hitachi. Transmission electron microscopy (TEM) was carried out to check the shape, size and polymer coating. A drop of the diluted sample was placed onto a TEM grid, filter paper was used to pull excess moisture off the sample, and further moisture was left to evaporate off under a heat lamp and observed by (H-7500)-Hitachi. In addition, the surface roughness, topographical maps and agglomerated particles morphology with HA conjugated Cur-HA NPs are compared to uncoated NPs by Atomic force microscopy (AFM). A Veeco Dimension 3100 AFM was set to tapping mode and used Nanoworld TESPA-10 POINTPROBE Silicon SPM-Sensor tips to generate three 20 × 20 µm surface scans of each sample. For each scan, a 512 × 512 matrix was generated along the *x*–*y* plane corresponding to surface topography.

### 2.5. FTIR Analysis of Hyaluronan Nano Conjugate System

The control PLGA, chitosan-PLGA, Hyaluronan conjugated particles were analyzed by FT-IR (FTIR 660-IR Varian). The IR spectra of the samples were obtained using the reflection transmission spectroscopy technique involving the use of an ARO (all reflective objective) lens. A total of 32 scans were run for each sample at a resolution of 4 cm^−1^ and the spectra recorded from 400 to 4000 cm^−1^. The spectral data was collected using the Resolution Pro^®^ software and the numerical values plotted for graphical representation.

### 2.6. Drug Encapsulation and Loading Measurements

Encapsulation and loading efficiencies were measured by the centrifugation method. The amount of curcumin entrapped within the nanoparticles was determined indirectly by measuring the free drug content in the supernatant after being centrifuged at 13,000 rpm at 4 °C for 15 min and quantified by photometric analysis at 436 nm by UV spectroscopy. Encapsulation efficiency was calculated using the equation below,

$$\frac{\mathrm{Amount}\;\mathrm{of}\;\mathrm{drug}\;\mathrm{in}\;\mathrm{nanoparticles}}{\mathrm{Initial}\;\mathrm{amount}\;\mathrm{of}\;\mathrm{drug}\;\mathrm{used}}\;\times \;100\;=\;\%\;\mathrm{Encapsulation}\;\mathrm{efficiency}$$

Subsequently, the drug loading in nanoparticles was determined directly by dissolving HA-CU-NPs in three pre-weighed eppendorfs. The three eppendorfs were then re-weighed and the weight of drug and polymer was noted. These eppendorfs were then centrifuged at 13,000 rpm at 4 °C for 15 min to pellet the product and the obtained supernatant was analyzed by photometric analysis at 436 nm by UV spectroscopy. Loading efficiency was calculated using the equation below,

$$\frac{\mathrm{Weight}\;\mathrm{of}\;\mathrm{drug}\;\mathrm{in}\;\mathrm{nanoparticles}}{\mathrm{Total}\;\mathrm{weight}\;\mathrm{of}\;\mathrm{nanoparticles}}\;\times \;100\;=\;\%\;\mathrm{Loading}\;\mathrm{efficiency}$$

### 2.7. In Vitro Drug Release Studies in Simulated GI Fluids

In vitro drug release experiments were carried out in a commercially available Slide-A-Lyzer^®^ MINI Dialysis Device (10K MWCO, 50 mL tubes). To each dialysis bag containing 45 mL of dissolution medium, 200 μL of nanoparticle suspension was added. The pH values were selected based upon the normal variation of gastrointestinal tract (GIT) in the stomach (pH ~ 1.5), to the colon (pH 6.8 to 7.8). Simulated gastric fluid (SGF) of pH 1.2 (using 0.1 N HCl and 2 g NaCl for 1 L) was prepared according to the United States pharmacopeia. For the first 2 h, the dissolution study was carried out in 45 mL of pH 1.2 HCl buffer, pepsin (144 mg) using 200 rpm at 37 ± 0.5 °C. Afterwards, the pH of the dissolution media was adjusted to pH 6.8 by 1M NaOH and addition of KH2PO4 (153 mg) and Na_2_HPO_4_·2H_2_O (232 mg), pancreatine (225 mg) to achieve the intestinal sink condition, the study was continued for up to 3 days, 1.5 mL sample was withdrawn from the release profile medium at pre-determined time intervals and centrifuged at 13,000 *g* for 15 min. Subsequently, a 1 mL sample from the supernatant was quantified by photometric analysis at 436 nm by UV Spectroscopy.

### 2.8. Nanoparticle Degradation Studies in Simulated Gastrointestinal Media

In vitro degradation studies of NPs were performed by treating them with simulated gastric (acidic), intestinal (basic) environments. 50 μL of CU-NPs was treated with 1 mL simulated gastric media (3.2 mg pepsin), incubated at 37 °C at 150 rpm for 2 h. Further, NPs were treated with 1 mL simulated intestinal fluids (5 mg pancreatin and 13.15 U hyaluronidase) for 5 h and 48 h followed by centrifugation of the samples at 13,000 rpm for 5 min. The external morphology of the extracted nanoparticle dispersion was analyzed by SEM.

### 2.9. Cell Cytotoxicity Assays

The colon epithelial like carcinoma cell lines (HT-29, Caco-2 cell model) mimic the intestinal epithelium layer, and constitute an effective in vitro cell line model for studying cytotoxicity, uptake, permeability studies etc. for colon-specific drug delivery systems. Human Caco-2 cells (Caco-2/HTB-37) (ATCC, Manassas, VA, USA) were grown in minimum essential media (MEM); Human HT-29 cells (HT-29/HTB-38) (ATCC, Manassas, VA, USA) were grown in basal media consisting of Dulbecco’s Modified Eagle’s Medium (DMEM) supplemented with 10% foetal calf serum (FCS) and 1% penicillin/streptomycin respectively. In all cases, cells were grown until 80%–90% confluent and washed three times by rinsing with Hanks’ balanced salt solution (HBSS) before all experiments. Cell (Caco-2, HT-29) metabolic activity after nanoparticles treatment: Caco-2, HT-29 cells (50 K cells to each well) were seeded in 48-well micro-plates in triplicate. An Alamar blue assay was performed to test the cell (Caco-2, HT-29) metabolic activity upon treating with prepared nanoparticles with Blank-NPs, CU-HA NPs (0.1, 1, 10 and 100 μg/mL) for 24 h. A Picogreen assay was performed to test the cell (Caco-2, HT-29) DNA quantification upon treating with prepared nanoparticles with Blank-NPs, CU-HA NPs (0.1, 1, 10 and 100 μg/mL) for 24 h. Live/Dead assay was performed to test the cell (Caco-2 colorectal carcinoma cell line) cytotoxicity upon treating with nanoparticles. 2 μM Calcien AM (CA) and 4 μM Ethidium Homodimer-1 (ET) staining solution were prepared in HBSS and warmed in dark and sterile conditions. 150 μL of CA/ET solution was added to each well and incubated for 30–45 min. The inverted-fluorescence microscope was used to image cells—for the Calcein, the FITC filter, and for Ethidium homodimer-1, the Texas Red filter was used.

### 2.10. Nanoparticles Uptake Studies on Colon Epithelial Like HT-29 Cell Line

Fluorescent curcumin loaded HA nanoparticles uptake studies were conducted by HT-29 (colorectal adenocarcinoma, epithelial, adherent type). Culture HT-29 cells in 8 well chamber slides (at a respective density of 1 × 10^5^ cells/well) for 24 h. The medium was removed and added with serum-free medium containing NPs (100 μg/mL Curcumin-HA NPs) for 3 h. After incubation for different time-periods, the cells were carefully rinsed with HBSS (3×) to eliminate excess NPs, which were not taken up by cells. Subsequently, the treated cells were harvested using trypsin (0.5 mL Trypsin+ 0.5 mL media), and transferred into a 2 mL eppendorf and centrifuged at 1800× *g* for 5 min. Fix cells with 4% PFA (500 uL for each eppendorf) for 10 min at room temp. Centrifuge at 1800× *g* for 5 min and remove the PFA. Add (300 μL) 0.1% Triton-X to permeabilize cells for 2 min at room temperature. Centrifuge at 1800× *g* for 5 min, and remove Triton-X. Wash the cells once with PBS and centrifuge at 1800× *g* for 5 min. Stain the cells with Rhodamine Phalloidin for 15 min (1:1000 in PBS dilution, for actin, cytoskeleton staining) and wash with PBS (2×); DAPI for 5 min (1:2000 in PBS dilution, for nucleus staining) wash with PBS (2×). After washing, cells were re-suspended in 30 μL PBS buffer, and kept at 4 °C until further analysis by Flow (Image stream and Canto), and with the separate set of experiments wherein the chamber slides are imaged by a high slide throughput Olympus VS120 Digital Scanner.

## 3. Results and Discussion

### 3.1. Synthesis, Physicochemical Characterization of Cur-HA Nanoconjugate System

**Figure 1:**
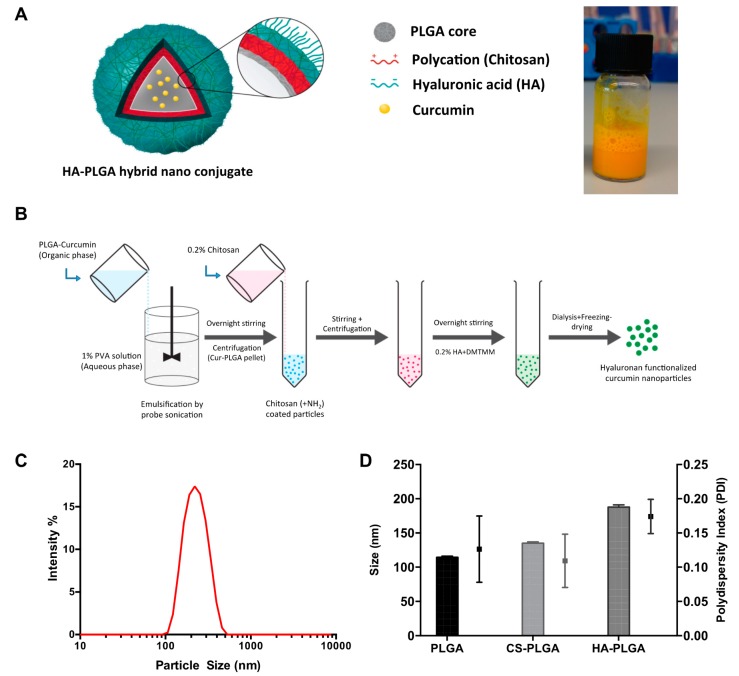
Concept figure of curcumin model drug loaded polymeric hybrid nanoparticles synthesis and characterization (**A**). Schematic of fabrication of the nanoconjugate system (**B**). Typical dynamic light scattering size intensity percentage graph (**C**). Size and Polydispersity index, PDI of drug core, chitosan coating and hyaluronan conjugation respectively (**D**).

In the present study, we have used a model fluorescent small drug molecule (curcumin) to design, fabricate, optimize and evaluate the colon-specific nanoconjugate system. The curcumin-loaded hyaluronan functionalized nanoparticle (Cur-HA NPs) system was successfully synthesized by the layer-by-layer approach with the emulsification-solvent evaporation method (Figure 1A,B). At each stage of the fabrication (chitosan coating, HA-conjugation), the nanoparticles were assessed for their size and polydispersity index (PDI) by dynamic light scattering (DLS), and consistency was checked for the different batches of the nanoparticles synthesized. The mean effective diameter of Cur-HA NPs detected by dynamic light scattering assay was around 220 nm (as shown in (Figure 1C). The sizes of the nanoparticles in the adsorption and coating process on core Cur-PLGA NPs were also determined using DLS, which exhibited an increase in size upon each coating (Figure 1D).

### 3.2. Morphological, Surface Charge, FT-IR Analysis of Cur-HA Nanoconjugate System

**Figure 2:**
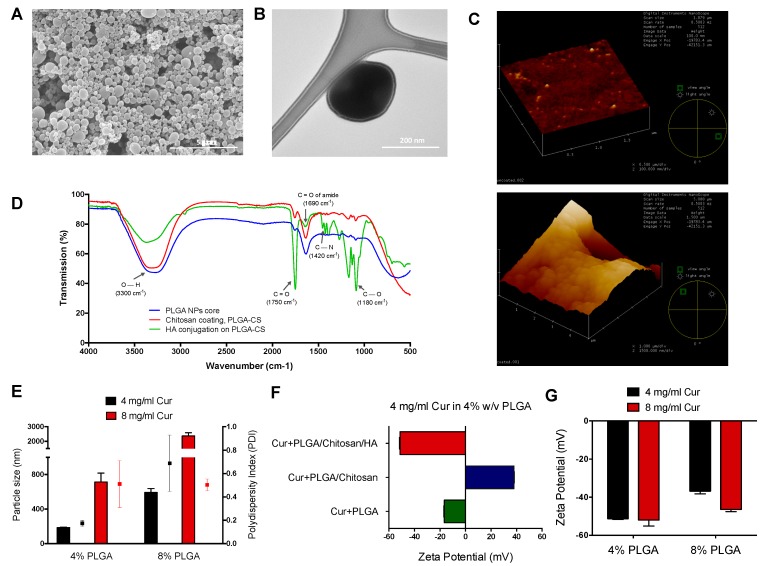
Morphological, physicochemical properties of curcumin loaded polymeric hybrid nanoparticles. Scanning electron microscopy image of HA conjugated NPs (scale bar = 5 μm) (**A**). Transmission electron microscopy image showing the coated layer of the individual nanoparticle (scale bar = 200 nm) (**B**). AFM images of uncoated, HA conjugated NPs (**C**). FT-IR graph showing the hyaluronan (-COOH) conjugation on PLGA-Chitosan (+NH2) core (**D**). Size and PDI of 4, 8 mg/mL drug to 4%, 8% polymer ratio (**E**). Change in surface zeta potential after each layer of coating with chitosan and hyaluronan conjugation respectively (**F**). Zeta potential of different groups of drug to polymer ratios (**G**).

The surface morphology of the particles was assessed by scanning electron microscopy (SEM), Transmission electron microscopy (TEM), which reveals that nanoparticles were spherical in shape, monodispersed and varied in size from 200–300 nm (Figure 2A,B). Atomic force microscopy revealed that there is a difference in size, surface roughness, and agglomerated particle morphology with HA conjugated Cur-HA NPs when compared to uncoated NPs (Figure 2C). The control PLGA, chitosan-PLGA, HA conjugated particles were analyzed by FT-IR to identify the chemical composition of the nanoparticle formulation system and the conjugation of HA, and to reveal any significant interactions between the materials in the formulation mixtures. FT-IR spectra for PLGA, Chitosan coated, and HA-conjugated system confirmed the presence of –C=O, –C–N groups on HA functionalized NPs (Figure 2D). Nanoparticle formulations of 4, 8 mg/mL Cur at 4%, 8% PLGA were assessed by DLS for their size and polydispersity index (PDI). The 4% PLGA 4 mg/mL Cur and 8 mg/mL Cur yielded 187.7 ± 3.35 nm and 714.2 ± 102.3 nm respectively for their size analysis. The PDI for 4% PLGA with 4 mg/mL Cur and 8 mg/mL Cur resulted in 0.174 ± 0.025 and 0.511 ± 0.201 respectively (Figure 2E), indicating for higher the drug-polymer concentrations there is an increase in size, heterogeneity of the formulation.

Cur loaded PLGA nanoparticles yielded an overall charge of −16.5 ± 0.4 mV, and further chitosan coating changed the surface charge to 38.0 ± 0.3 mV as the amines (+) from chitosan formed a layer on the core particles. HA-conjugated Cur loaded nanoparticles yielded an overall charge of −51.3 ± 0.4 mV (Figure 2F). More specifically, 4% PLGA at 4 mg/mL Cur and 8 mg/mL Cur yielded −51.7 ± 0.4 mV and −52 ± 3.2 mV respectively, and other ratios of nanoparticles at 8% PLGA 4 mg/mL Cur and 8 mg/mL Cur yielded −36.8 ± 1.5 mV and −46.4 ± 1.1 mV respectively of their surface charge analysis after the HA-conjugation of nanoparticle preparation (Figure 2G).

### 3.3. Drug Content, In Vitro Simulated GI Fluids Effect on Degradation and Drug Release

**Figure 3:**
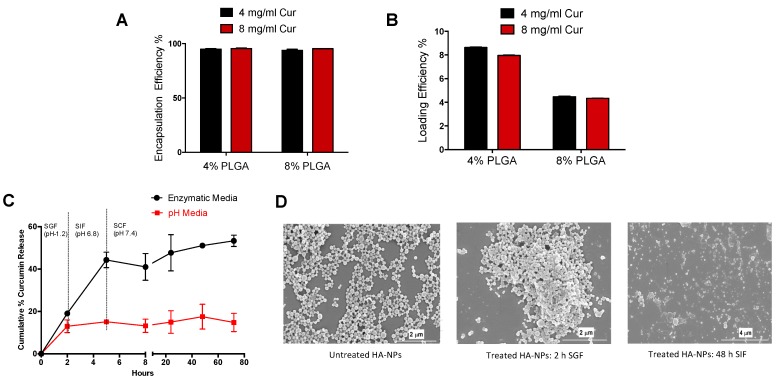
Curcumin encapsulation, loading efficiencies and drug release studies in simulated gastrointestinal fluids. Encapsulation and loading efficiencies of different polymer to drug ratios (**A**,**B**). In vitro curcumin release profile in pH and simulated gastric, intestinal fluids (**C**). Scanning electron microscopy images of in vitro particle degradation of untreated, 2h simulated gastric fluid treated, 48h simulated intestinal fluid treated NPs (**D**).

To analyze the variables affecting drug encapsulation and loading, HA-conjugated nanoparticles loaded with curcumin were fabricated using two concentrations of curcumin (4 mg/mL & 8 mg/mL) and two concentrations of PLGA (4% and 8%). A lower drug concentration (4 mg/mL) and a lower polymer concentration (4% PLGA) resulted in high drug-loading efficiency (8.6%) among the formulations tested (Figure 3B). In contrast, the encapsulation efficiency was not substantially affected by the drug and polymer concentrations (Figure 3A).

The in vitro release profile of curcumin from HA-conjugated nanoparticle system was investigated in simulated gastrointestinal media (gradually pH changing media and enzymatic media) to mimic simulated stomach (pH 1.2 with pepsin) and intestinal conditions (small intestine: pH 6.8 with pancreatine; colon: pH 7.2 with β-glucuronidase and hyaluronidase). In pH media, less than 20% of curcumin was released over 72 h from nanoparticles. However, in enzymatic media there was a burst release in the first 5 h with 40% release of curcumin, which was sustained over 72 h with a mean release of 53%. With the addition of colonic enzymes such as β-glucuronidase and hyaluronidase to degrade the HA and chitosan layer, there was a substantial increase in release of curcumin from nanoparticles. Alone pH media does not allow the release of curcumin from the nanoparticle system and is not biologically relevant to represent an in vivo release setting of oral drug delivery system (Figure 3C).

In addition, the scanning electron microscopy images of the nano drug delivery system after incubation with simulated gastric media (2 h exposure) and simulated intestinal media (48 h exposure) reveals how the drug-polymeric hybrid system degradation affects nanoparticle structures (Figure 3D). Compared to untreated Cur-HA NPs, enzymatic buffer media induced surface changes in the Cur-HA NPs. In acidic conditions, the Cur-HA-NPs retained their shape and were seemingly unaffected by the acidic enzymatic media; this may have been due to HA having a protective effect on the particles, allowing them to remain stable in acid conditions. The morphology of the particles started to change as the pH was changed and as the enzymes pancreatin and hyaluronidase were added. Although the specific underlying degradation mechanism remains complex, the polymeric system, upon exposure to the diffusion of GI fluids into the nano cargo, and the enzymatic milieu might help in the degradation of the delivery system in vitro.

### 3.4. Cytotoxicity Studies of Cur-HA-NPs on Colon Epithelial-Like Cells

**Figure 4:**
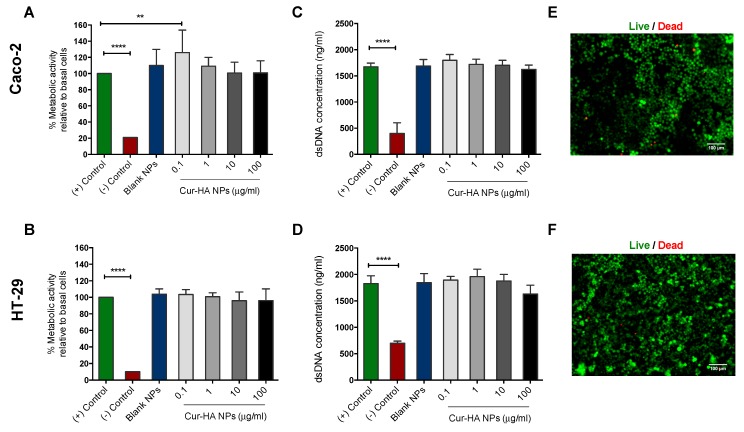
Cytotoxicity studies of Cur-HA-NPs on colon epithelial like cells (Caco-2, HT-29)**.** Alamar Blue assay of blank, Cur-HA NPs (0.1, 1, 10 and 100 μg/mL), (Media alone-positive control) (DMSO treated- negative control) treatment for cell metabolic activity on Caco-2 and HT-29 cell lines (**A**,**B**). Picogreen assay of blank, Cur-HA NPs (0.1, 1, 10 and 100 μg/mL) treatment for cell proliferation on Caco-2 and HT-29 cell lines (**C**,**D**). Live/Dead assay images of Caco-2, HT-29 cells after 24 h nanoparticle treatments at 10× magnification (**E**,**F**). The results are expressed for each treatment as (*n* = 3); data are expressed as the mean ± SD. P values were determined by one-way ANOVA with Dunnett’s post hoc test to determine differences between treatments and control group; ** *p* < 0.01 for control vs. 0.1 ug/mL Cur-HA NPs in the Caco-2 cells, **** *p* < 0.0001 for (+) control vs. (−) control respectively. No significant differences were noted in the HT-29 cells.

The cytocompatability of cells is a primary concern in the efforts to develop new oral drug delivery systems. To evaluate the metabolic activity, proliferation, and cytotoxicity of the prepared nanoparticles, colon epithelial-like carcinoma cells (Caco-2 and HT-29) were treated with various NPs concentrations, examined by Alamar Blue, PicoGreen and Live/Dead assays respectively. Alamar Blue assay showed the effect of blank-HA NPs, Cur-HA NPs at various concentrations (0.1, 1, 10 and 100 μg/mL) on the metabolic activity of colon epithelial-like cells (Caco-2 and HT-29). The results in (Figure 4A,B) reveal that both blank-HA NPs, Cur-HA NPs show almost no toxicity to Caco-2 and HT-29 cells, indicating that both blank-HA NPs, Cur-HA NPs are suitable biocompatible nano-carriers to deliver any loaded hydrophobic therapeutics orally to the colon. There is no significant difference in the percentage cell viability between the treatment groups. In contrast, cell viability is significantly inhibited when treated with cell culture DMSO (negative control).

The PicoGreen assay on the Caco-2, HT-29 cell lines show no significant decrease in dsDNA concentration after blank-HA NPs, Cur-HA NPs treatment compared to control. There was a slight decrease in dsDNA concentration in Cur-HA NPs at 100 μg/mL in contrast to the control (Figure 4C,D). The Live/Dead assay images showed that blank-HA NPs, Cur-HA NPs treatments did not significantly alter cell viability in Caco-2 or HT-29 colon epithelial like carcinoma cell lines (Figure 4E,F). Thus, in overall terms, Cur loaded HA-conjugated nanoparticle system treatments did not affect cell cytotoxicity, as the components we used are generally recognized as safe (GRAS) by the U.S. Food and Drug Administration (USFDA), which are generally safe for oral consumption, inexpensive, easily available in large quantities and can be readily translated certainly into clinic.

### 3.5. Effect of HA Functionalization on HT-29 Colon Epithelial-Like Cell Interactions

**Figure 5:**
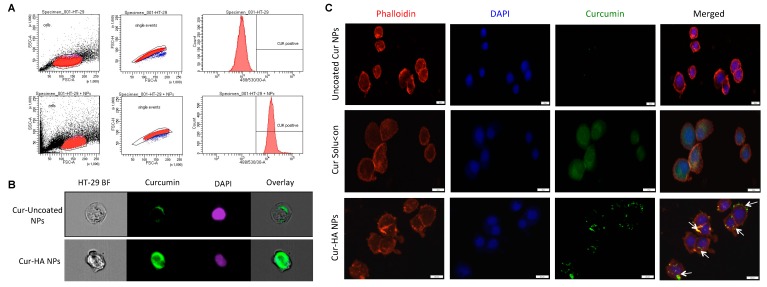
Hyaluronan functionalization increases the nanoparticles interaction, uptake in HT-29 cells. Flow cytometry histogram shows alone HT-29 cells, HT-29 cells treated with Cur-HA-NPs signal after 3 h incubation (**A**). Representative ImageStream flow cytometry images show HA functionalized particles has effective uptake in HA-29 cells (**B**). Representative slide scanner images of uncoated Cur NPs, Cur solution and Cur-HA NPs after 3 h incubation, HA functionalized NPs showing more cellular interactions and uptake compared to uncoated NPs; nuclei (blue) stained with DAPI, cytoskeleton (red) stained with rhodamine-phalloidin, curcumin (green) (**C**).

Epithelial cellular interaction is absolutely vital on the intestinal wall surfaces for an effective therapy especially in IBD or colon cancer. In the present study, we have used a small molecular fluorescent therapeutic compound curcumin to investigate the cellular level interactions and uptake behavior. The colon epithelial-like carcinoma cells (HT-29) was treated for three hours with Cur-HA-NPs (Cur, 100 μg/mL). As expected, the untreated control cells have shown no fluorescence signal, whereas a clear positive signal for curcumin with cells was observed by flow cytometry (Figure 5A). Further to confirm whether the functionalized HA nanosystem could promote cellular interactions and uptake, we incubated HT-29 cells with uncoated-cur NPs, Cur-HA NPs respectively and curcumin solution as a positive control. The HA functionalized particles showed favorable interactions on the cell surface and uptake by ImageStream flow cytometry analysis (Figure 5B) and slide scanner imaging (Figure 5C). Importantly, the presence of HA on the surface of the NPs allows better cellular interactions than that of uncoated NPs. This result emphasizes a possible selectivity of the system towards inflamed colonic epithelium or colon cancer cells in heterogeneous microenvironments via HA receptor-mediated endocytosis (CD44, RHAMM, TLRs), which are more overexpressed in those disease conditions than normal cells [22,32,33,34].

## 4. Conclusions

In the development of advanced oral drug delivery systems to the colon, our aim is to enhance the targetability and local availability of the drug while minimizing the associated drug side effects [4,35]. Currently there is considerable promise in oral nano/microparticle drug delivery systems to target the inflamed mucosa or cancer for more effective IBD and associated colon cancer therapies. Reducing the size of the particles, surface functionalization with targeted ligands also provides additional advantages. Nanoparticles allow for a certain amount of protection for the entrapped compound during GI passage, however, polymeric particles alone, such as PLGA, are unable to resist release within the small intestines, and an uncontrollable drug release frequently occurs before particles arrive at their intended destination. A few recent disease-specific targetability methods use ligand functionalization to attract the drug cargo to specific surface receptors, proteins and adhesion molecules at the disease site; however, oral administration of these systems encounters challenges from acidic and enzymatic degradation.

In conclusion, we report a strategy that uses a HA-functionalized polymeric hybrid nanoconjugate system fabricated from GRAS reagents for enhanced drug absorption with controlled drug release and increasing local drug bioavailability in the colon lumen. As expected, Cur-HA NPs could encapsulate the fluorescent small drug molecule-curcumin and exhibited suitable physicochemical characteristics (size, polydispersity and surface charge) for colonic delivery. The developed Cur-HA NPs were spherical in shape, with a uniform size and a smooth surface homogeneous nature. NPs with 4% PLGA, 4% mg/mL Cur demonstrated an acceptable particle size with low PDI, good encapsulation efficiency, and a higher zeta potential. The in vitro enzymatic degradation of nanoparticles exhibited an appropriate degradation profile for nanoparticles, showing that particles are degrading and that their morphology changes over time in simulated gastric and intestinal fluids. Layer-by-layer coating of the polysaccharides (chitosan, HA) protected the drug release in the upper GIT, and the release of the drug was high in the colonic regions with a prolonged manner. In addition, in vitro cell culture studies on Caco-2 and HT-29 colon carcinoma cell lines showed that nanoparticle treatment did not affect metabolic activity or cytotoxicity, as the components used in the fabrication process are generally recognized as safe (GRAS). Furthermore, HA functionalization enhanced the adhesion, interaction, and uptake of the particles on colon cells in vitro. Overall, our results suggest that this proof-of-concept could potentially be utilized to deliver any hydrophobic therapeutics to the colon that could produce potential improvements in colon inflammation, and cancer treatments. Further studies are needed to investigate the abilities of the NPs system with the beneficial therapeutic molecule, and to examine the therapeutic efficiency in in vivo models.
